# Identification of a linear B-cell epitope on the *Schistosoma japonicum* saposin protein, SjSAP4: Potential as a component of a multi-epitope diagnostic assay

**DOI:** 10.1371/journal.pntd.0010619

**Published:** 2022-07-11

**Authors:** Yi Mu, Catherine A. Gordon, Remigio M. Olveda, Allen G. Ross, David U. Olveda, Jessica M. Marsh, Donald P. McManus, Pengfei Cai

**Affiliations:** 1 Molecular Parasitology Laboratory, QIMR Berghofer Medical Research Institute, Brisbane, Australia; 2 Research Institute for Tropical Medicine, Department of Health, Manila, Philippines; 3 Research Institute for Rural Health, Charles Sturt University, Orange, Australia; 4 Department of Pathology, JONELTA Foundation School of Medicine, University of Perpetual Help Rizal, Manila, Philippines; 5 The School of Biomedical Sciences, Queensland University of Technology, Brisbane, Australia; University of Passo Fundo: Universidade de Passo Fundo, BRAZIL

## Abstract

**Background:**

*Schistosoma japonicum* is one of three major species of blood flukes causing schistosomiasis, a disease, which continues to be a major public health issue in the Philippines. SjSAP4, a member of a multigene family of saposin-like proteins, is a recognized immunodiagnostic biomarker for schistosomiasis japonica. This study aimed to identify linear B-cell epitopes on SjSAP4 and to validate their potential as components of a multi-epitope assay for the serological diagnosis of schistosomiasis japonica.

**Methodology:**

SjSAP4-derived peptides were expressed as GST-peptide-fused proteins and these were Western blot probed with human serum samples from *S*. *japonicum* Kato-Katz (KK)-positive individuals and uninfected controls. A core epitope was further identified by Western blotting through probing a series of truncated peptides with the schistosomiasis patient sera. The diagnostic performance of the core epitope-containing peptides and the full-length SjSAP4 was evaluated by enzyme-linked immunosorbent assay (ELISA) using a panel of sera collected from subjects resident in a schistosomiasis-endemic area of the Philippines.

**Main findings:**

As a result of the peptide mapping, one peptide (P15) was found to be highly immunogenic in the KK-positive individuals. We subsequently showed that -S^163^QCSLVGDIFVDKYLD^178^- is a core B-cell epitope of P15. Subsequent ELISAs incorporating SjSAP4, SjSAP4-Peptide and SjSP-13V2-Peptide showed a sensitivity of 94.0%, 46.0% and 74.0%, respectively, and a specificity of 97.1%, 100% and 100%, respectively. Notably, complementary recognition of the B-cell epitopes (SjSAP4-Peptide and SjSP-13V2-Peptide) was observed in a subset of the KK-positive individuals. A dual epitope-ELISA (SjSAP4-Peptide + SjSP-13V2-Peptide-ELISA) showed a diagnostic sensitivity of 84.0% and a specificity of 100%.

**Conclusions/Significance:**

In this study, -S^163^QCSLVGDIFVDKYLD^178^- was identified as a dominant linear B-cell epitope on SjSAP4. This peptide and the complementary recognition of other B-cell epitopes using sera from different KK-positive individuals can provide the basis of developing a multi-epitope assay for the serological diagnosis of schistosomiasis.

## Author summary

The recent road map (2021–2030) released by WHO for controlling or eliminating neglected tropical diseases (NTDs) highlights diagnostics as a major focus. Development and deployment of accurate, affordable and field-friendly diagnostics/surveillance tools will be crucial for the control and elimination of schistosomiasis. Multi-epitope chimeric antigens, constructed based on linear B-cell epitopes identified from highly antigenic antigens, may achieve not only an equivalent or superior diagnostic performance compared to the parent immunogens but also exhibit more optimal physicochemical properties. However, to date, only a limited number of linear B-cell epitopes have been identified for the serological diagnosis of schistosomiasis. In this study, we identified a linear B-cell epitope (-S^163^QCSLVGDIFVDKYLD^178^-) on SjSAP4, a recognized immunodiagnostic biomarker for schistosomiasis japonica, and validated its potential as a component of a multi-epitope assay for the serological diagnosis of schistosomiasis japonica. Notably, differential recognition of B-cell epitopes (SjSAP4-Peptide and SjSP-13V2-Peptide) was observed in a subset of subjects positive by the Kato-Katz technique for the disease. A dual epitope-ELISA (SjSAP4-Peptide + SjSP-13V2-Peptide-ELISA) showed a superior diagnostic performance (84.0% sensitivity and 100% specificity) than individual-epitope ELISAs. The findings in this study provide support for the development of multi-epitope antigen-based diagnostics for schistosomiasis.

## Introduction

Schistosomiasis, an important neglected tropical disease (NTD) caused by agents of the genus *Schistosoma*, remains a serious public health problem afflicting over 230 million people in 78 countries [1]. Most human schistosomiasis arises from three major schistosome species, i.e., *Schistosoma japonicum* (Sj), *S*. *mansoni* (Sm) and *S*. *haematobium* (Sh), of which the zoonotic species *S*. *japonicum* is prevalent in the Philippines, the People’s Republic of China and small pockets of Indonesia [2]. Mass drug administration (MDA) is the cornerstone of the current control strategy for schistosomiasis [3]. However, MDA on its own is insufficient to provide long-term sustainable control of the disease if no additional integrated interventions are implemented [3, 4]. This is especially the case in a country like the Philippines, where there is wide-spread distribution of its *Oncomelania hupensis quadrasi* snail intermediate hosts and extensive numbers of domestic mammals (e.g., bovines) that act as reservoirs for human infection. In this post-MDA era, affordable and accurate diagnostic tools for rapid mapping of schistosomiasis in the context of integrated control programmes are urgently needed [5–8].

Traditional parasitological detection methods, such as the Kato-Katz (KK) coproparasitological test and urine filtration, although showing high specificity, exhibit low sensitivity, particularly when applied in areas with reduced prevalence/disease intensity [9–12]. Serology, including antibody-detection (AbD) and antigen-detection (AgD) are usually cost-effective tools for large-scale screening to monitor schistosomiasis control efforts. AgD-based methods, mainly through detecting circulating anodic antigen (CAA) or circulating cathodic antigen (CCA), in the format of lateral flow assays in urine samples, provide rapid diagnostic test readouts [13, 14]; yet accumulating evidence demonstrates limitations of the point of care (POC)-CCA assay in detecting low intensity infections [12, 15–17]. Previous AbD methods primarily using crude extracted antigens (e.g. soluble egg antigen (SEA)), although showing sufficient sensitivity, presented wide-scale cross-reactivity with antibodies generated against antigens released by other helminth species in target cohorts. The identification of highly specific schistosome antigens with a high level of sensitivity is thus a prerequisite for developing AbD-based serological diagnostics [18].

Recently, a group of novel schistosome antigens with high immunogenicity were identified through high-throughput immunomics [19–22], including members of a multigene family of saposin-like proteins [20, 23]. In *S*. *japonicum*, this multigene family is composed of at least 15 members (SjSAPLP1 to SjSAPLP15) [23]. Some schistosome saposin members have been suggested to be involved in lysis of ingested red cells in the gut of the parasite [24], and have been identified in the worm vomitus [25], which may be continuously released into the host circulatory system, stimulating the host to produce a strong immune response. SjSAP4, a member of a multigene family of saposin-like proteins, showed an unprecedented diagnostic performance in enzyme-linked immunosorbent assay (ELISA) for detecting infected individuals in cohorts from Asian schistosomiasis-endemic areas, both in China and the Philippines [23, 26]. The protein contains a single saposin-B type (Sap-B) domain at the C-terminus that has a characteristic set of three disulfide bridges formed by six cysteines [23]. Transcriptional analysis revealed that SjSAP4 is highly expressed in cercariae, schistosomula and adult worms but not in eggs [26]. In adult worms, SjSAP4 is abundantly expressed in the gastrodermis [23].

Incorporating multi-epitope antigens may further improve the sensitivity and specificity of AbD-based serology tests. In the schistosome research area, most studies on epitope identification were carried out for vaccine development [27–29] with reports on the identification of B-cell epitopes for diagnostic purposes being rare. Recently, linear epitopes recognized by sera from patients with schistosomiasis were identified on the saposin-like protein, SjSP-13, and their potential as serological markers for schistosomiasis japonica demonstrated [30]. In this study, we aimed to identify linear B-cell epitopes on the promising diagnostic antigen, SjSAP4, and validate their diagnostic performance in the detection of *S*. *japonicum* infection with serum samples obtained from a cohort recruited from schistosomiasis-endemic areas in the Philippines.

## Materials and methods

### Ethics statement

The human research ethical approval for the study was granted by the Institutional Review Board of the Research Institute for Tropical Medicine (RITM), Department of Health, Manila, the Philippines and the Human Research Ethics Committee, QIMR Berghofer Medical Research Institute (QIMRB), Brisbane, Australia (Ethics Approval: P524). Written informed consent form was received from each study participant (for those under the age of 15 years, written informed consent form was received from their legal guardians).

### Study cohort and human sample collection

The human cohort subjects were recruited from areas moderately endemic for schistosomiasis japonica in the municipalities of Laoang and Palapag, Northern Samar province, the Philippines. Additional information on the study population is available in previous reports [31–33]. For each participant, blood samples (10 mL) were collected with tubes (10 mL silica vacutainers) and serum samples were then obtained by centrifugation, and stored at 2–8°C. The serum samples were transported to the Research Institute for Tropical Medicine (RITM), and stored at -80°C. A subset of samples was subsequently shipped to QIMR Berghofer Medical Research Institute (QIMRB), Australia, on dry ice. Serum samples of healthy human subjects (n = 35) were collected from Heilongjiang Province, China, a non-endemic area for schistosomiasis and served as controls.

### Parasitological detection (Kato-Katz)

Each individual from the study cohort provided two stool specimens from which three KK thick smear slides were prepared for each specimen. Slides were examined by experienced laboratory technicians under a light microscope. The intensity of infection is presented as the number of eggs per gram of faeces (EPG). A subset of KK-positive individuals (n = 50) were used for epitope identification and diagnostic performance evaluation (S1 Table).

### B-cell epitope identification via peptide mapping

The SjSAP4 (GenBank accession number ON241030) protein contains 197 amino acids (AA), of which 18 AA at the N-terminus are predicted to be a signal peptide. SjSAP4, without the signal peptide (179-AA in length), was divided into 17 peptides with a length of 20 AA per peptide, except for the final peptide, which is 19-AA in length. There were 10-overlapped AA between any two adjacent peptides. The DNA sequences coding each peptide were amplified from a pET28a-SjSAP4 plasmid constructed in a previous study [26]. After digestion with *BamH*I and *Xho*I, the resulting DNA fragments were cloned into the pGEX-4T-1 vector. The expression of GST-peptide proteins in the *E*. *coli* BL21 strain was induced by 1 mM Isopropyl-D-1-thiogalactopyranoside (IPTG). The recombinant GST was purified on Glutathione Agarose (Thermo Fisher Scientific Australia Pty Ltd, Scoresby, VIC, Australia) according to the manufacturer’s instructions. Recombinant SjSAP4 was purified using Ni-NTA agarose (QIAGEN, Hilden, Germany) according to the manufacturer’s instructions as previously described [26]. The protein concentration was determined using the Qubit Protein Assay (Thermo Fisher Scientific Australia Pty Ltd, Scoresby, VIC, Australia). B-cell epitopes were then identified by Western blot through probing GST-peptide proteins with serum samples from KK-positives and healthy controls.

### B-cell epitope prediction and validation

B-cell epitope prediction was performed for SjSAP4 using the online predictors ABCpred [34] and BepiPred [35]. Based on the prediction results of the two servers, three peptides that potentially contained a B-cell epitope were selected for validation. These peptides were expressed as GST-peptide-fused proteins through cloning using the pGEX-4T-1 vector as described above and validated by Western blot.

### Identification of core B-cell linear epitope

To determine the core B-cell linear epitope, the original B-cell epitope-containing peptide was truncated into a series of peptides, which were further cloned into the pGEX-4T-1 vector and expressed as GST-peptide-fused proteins as described above. The core B-cell linear epitope was further identified by Western blot. All the primer sequences used in this study are listed in S2 Table.

### Western blotting

Sera from KK-positive individuals and healthy controls were used for epitope screening. Sera from KK-positives were incubated with purified GST protein to remove the corresponding antibodies before use. A mixture of 100 μl human serum and 25 μl purified GST (0.4 mg/ml) in 4 ml PBS (pH 7.4) was rocked for 5 h at room temperature. Pre-incubated serum samples were stored at -80°C until use. Proteins were separated on 12% Mini-PROTEAN TGX Precast Protein Gels (Bio-Rad, Hercules, CA, USA), and transferred to polyvinylidene difluoride membranes (Millipore, Bedford, MA, USA). The blots were blocked in Odyssey Blocking Buffers (Li-COR Biosciences, Lincoln, NE, USA) for 1 hour at room temperature. The membranes were then incubated with anti-GST tag rabbit polyclonal antibody (1:2,000) (Merck Pty Ltd, Bayswater, VIC, Australia), and sera from KK-positives and healthy controls (1:100). After incubation with IRDye 800CW goat Anti-Rabbit IgG (H+L) antibodies (1,20,000) or IRDye 800CW goat Anti-human IgG (H+L) antibodies (1:20,000) (Li-COR Biosciences, Lincoln, NE, USA), fluorescence signals were detected using an Odyssey CLx Imaging System (Li-COR Biosciences, Lincoln, NE, USA).

### Structure prediction and three-dimensional (3D) visualization of core epitopes

Peptide sequences of all saposin domains from SjSAP4, SjSP-13 variant 1 (SjSP-13V1) (GenBank accession number ON241031) and SjSP-13 variant 2 (SjSP-13V2) (GenBank accession number ON241032) were aligned by ClustalX 2.0. The structure-based alignment of these saposin domains was generated by the online program ESPript 3 [36] using the crystal structure of human saposin A (PDB codes 2dob) as a template. Prediction of the 3D structures of the SjSAP4 and SjSP-13V2 saposin domain was performed by the online service Phyre 2.0 [37]. The positional structure of the epitopes within the SapB domain of SjSAP4 and SjSP-13V2 was represented with the PyMOL Viewer program (DeLano Scientific, San Carlos, CA, http://www.pymol.org/). The 3D structure of SjSAP4 (without signal peptide) was predicted by the online algorithm Robetta (https://robetta.bakerlab.org).

### ELISA

Three peptides originating from the Philippines strain of *S*. *japonicum* (SjP) (a peptide from the 23-kDa integral membrane protein of *S*. *japonicum* (Sj23); -TSFHCCGVKGPDDYKGNVPASCK-, which is homologous to the B-cell epitope, P14 of Sm23 [38], SjSP-13V2 peptide: -EWKNKCLDVTDNLPEKIIQFANHMNIL- [30]; and SjSAP4 peptide: -**K**QSQCSLVGDIFVDKYLDM**K**- (a positively charged amino acid, lysine was added to the ends of the 18-AA SjSAP4 peptide Q^162^-M^179^ to increase solubility)) with a purity >95% were synthesized commercially (GenicBio Limited, Shanghai, China). ELISA was performed as described previously [26]. MaxiSorp high protein-binding capacity 96-well ELISA plates (Nunc, Roskilde, Denmark) were coated with SjSAP4 (1 μg/ml in coating buffer; 100 μl per well) or single peptide (5 μg/ml in coating buffer; 100 μl per well) or dual peptides (2.5 μg/ml of each peptide in coating buffer; 100 μl per well) overnight at 4°C. Wells were blocked with PBST (Phosphate-buffered saline, pH 7.5 with 0.05% Tween-20) containing 1% (w/v) BSA for 1 h at 37°C. Serum samples, diluted 1:100 with blocking buffer, were added to the wells and the mixtures were incubated for 1 h at 37°C. A mouse monoclonal anti-human IgG (Fc specific)-biotin antibody (Sigma-Aldrich Co, MO, USA) was then added as secondary antibody (1:20,000, 100 μL/well), followed by adding Streptavidin-HRP (BD Pharmingen, CA, USA) (1:10,000, 100 μL/well) to detect antibodies bound to SjSAP4 and peptides. Plates were washed with PBST for 5 times after each step. Enzymatic reactions were developed by adding 100 μl TMB substrate to each well and terminated by stop reagent (50 μl 2N H_2_SO_4_ per well). The optical density values at 450 nm (OD_450_) were measured with a POLARstar OPTIMA multidetection microplate reader (BMG LABTECH, VIC, Australia). Duplicate ELISA readings were undertaken. The cut-off value for a positive IgG response was set at 2.1 times the mean OD_450_ value of serum samples from healthy controls.

### Statistical analysis

Statistical analysis was performed with GraphPad Prism Version 8 for windows (GraphPad Software, Inc., San Diego, CA, USA). All the ELISA data are expressed as the mean ± standard error (SE). Relative quantification analysis on the recognition of different truncated peptides was performed with student’s *t*-test. For ELISA assays, the difference in OD_450_ between KK-positives and healthy controls was analyzed by the Mann-Whiten *U*-test. Diagnostic accuracy was evaluated by receiver operating characteristic (ROC) curve analysis. The area under the ROC curve (AUC) was calculated to assess the overall diagnostic performance. A *P*-value < 0.05 was considered statistically significant.

## Results

### Identification of linear B-cell epitopes on SjSAP4 by peptide mapping

**Fig 1:**
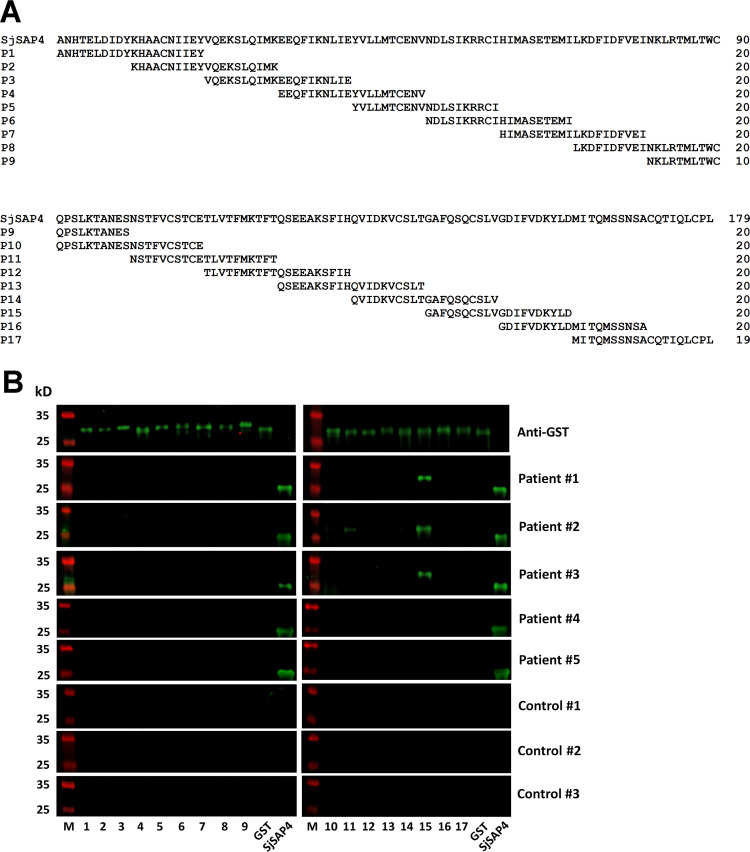
Identification of linear B-epitopes on SjSAP4. **(A)** Peptide design for epitope mapping. SjSAP4 without the signal peptide (179-AA in length) was divided into 17 peptides, including 16 peptides (P1 –P16) 20-AA in length and another peptide, P17, 19-AA in length. Ten AA were overlapped between any two adjacent peptides. **(B)** Western blot analysis showed that P15 was recognized by sera obtained from three out of five confirmed schistosomiasis japonica patients. M, Pre-stained protein ladder. Lane 1–17, GST-peptide fusion proteins GST-P1 –GST-P17, respectively. Recombinant SjSAP4 protein was used as a positive control. The serum samples from KK-positive individuals were pre-incubated with purified GST protein.

SjSAP4, without signal peptide, 179-aa in length, was divided into 16 20-AA peptides (P1-P16) and a 19-AA peptide (P17), with 10-overlapped AA between the two adjacent peptides (Fig 1A). These peptides were expressed as GST-peptide fusion proteins, which were confirmed by Western blot with an anti-GST antibody (Fig 1B). The recognition between the 17 SjSAP4-derived peptides and serum samples from *S*. *japonicum* KK-positive individuals was detected by Western blot analysis. Fig 1B shows that P15 (G^159^AFQSQCSLVGDIFVDKYLD^178^) could be recognized by serum samples from patient #1, #2, and #3, but not #4 and #5; while P11 was only weakly recognized by the serum sample from patient #2. None of the 17 peptides were recognized by the serum samples from the three healthy control individuals.

### Linear B-cell epitope prediction and validation on SjSAP4

Linear B-cell epitopes on SjSAP4 were predicted by the online prediction methods BepiPred and ABCPred. Based on the prediction results, three peptides PP1, PP2 and PP3 containing a potential B-cell epitope were selected for testing (S1 Fig). Based on the results of peptide mapping, P11 was weakly recognized by the serum sample from patient #2. We considered whether this peptide was a truncated B-cell epitope and if the charged amino acid E^107^ on the upwards of the P11 was involved in the antibody recognition. Consequently, an additional peptide PP4 (S1 Fig), comprising a combination of P10 and P11, was selected for validation. Western blot analysis showed that PP1 and PP4 were weakly recognized by the serum sample from patient #2 (S1 Fig).

### Identification of core epitope within P15

**Fig 2:**
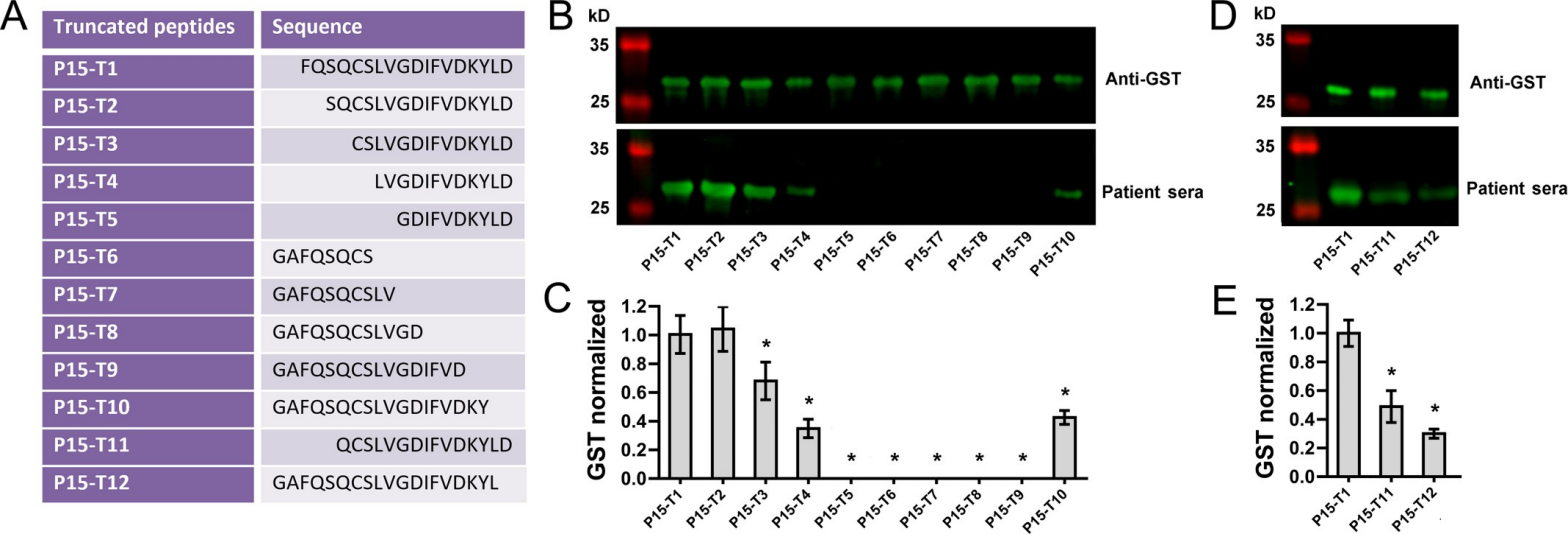
Identification of the core epitope on P15. **(A)** A panel of truncated peptides spanning the P15 region were expressed as GST fusion proteins. **(B)** Western blotting and **(C)** relative quantification analysis determined the recognition of the ten truncated peptides (P15-T1 – P15-T10) by pooled patient sera. Data are represented as the mean ± SD from three different assays (* *P* < 0.05 when compared with P15-T1 using the student’s *t*-test). **(D)** Western blot assay and **(E)** relative quantification analysis revealed a significant impaired binding of P15-T11 and P15-T12 when compared with P15-T1 using the student’s *t*-test. Data are represented as the mean ± SD from three different assays (* *P* < 0.05).

To define the core epitope on P15, a panel of truncated peptides spanning P15 were designed (Fig 2A). Of the ten progressive truncations of two AA of peptide P15 (P15-T1 – P15-T10), relative quantification analysis revealed significantly impaired recognition of P15-T3, P15-T4 and P15-T10 by the pooled patient sera, while P15-T5, P15-T6, P15-T7, P15-T8, and P15-T9 were not recognized by the serum pool (Fig 2B and 2C). The results thus indicated an impaired binding to antibody when the peptide lost S^163^Q^164^ at the N-terminal and L^177^D^178^ at the C-terminal. Western blotting and the followed relative quantification analysis revealed significantly impaired binding of another two truncated peptides P15-T11 and P15-T12 (Fig 2D and 2E), indicating that S^163^ and D^178^ are the N-terminus and C-terminus of the core epitope, respectively. The core epitope on P15 is thus a 16-AA peptide S^163^QCSLVGDIFVDKYLD^178^, spanning positions S^163^ to D^178^.

### SapB domain sequence alignment and 3D visualization of immunogenic epitopes

**Fig 3:**
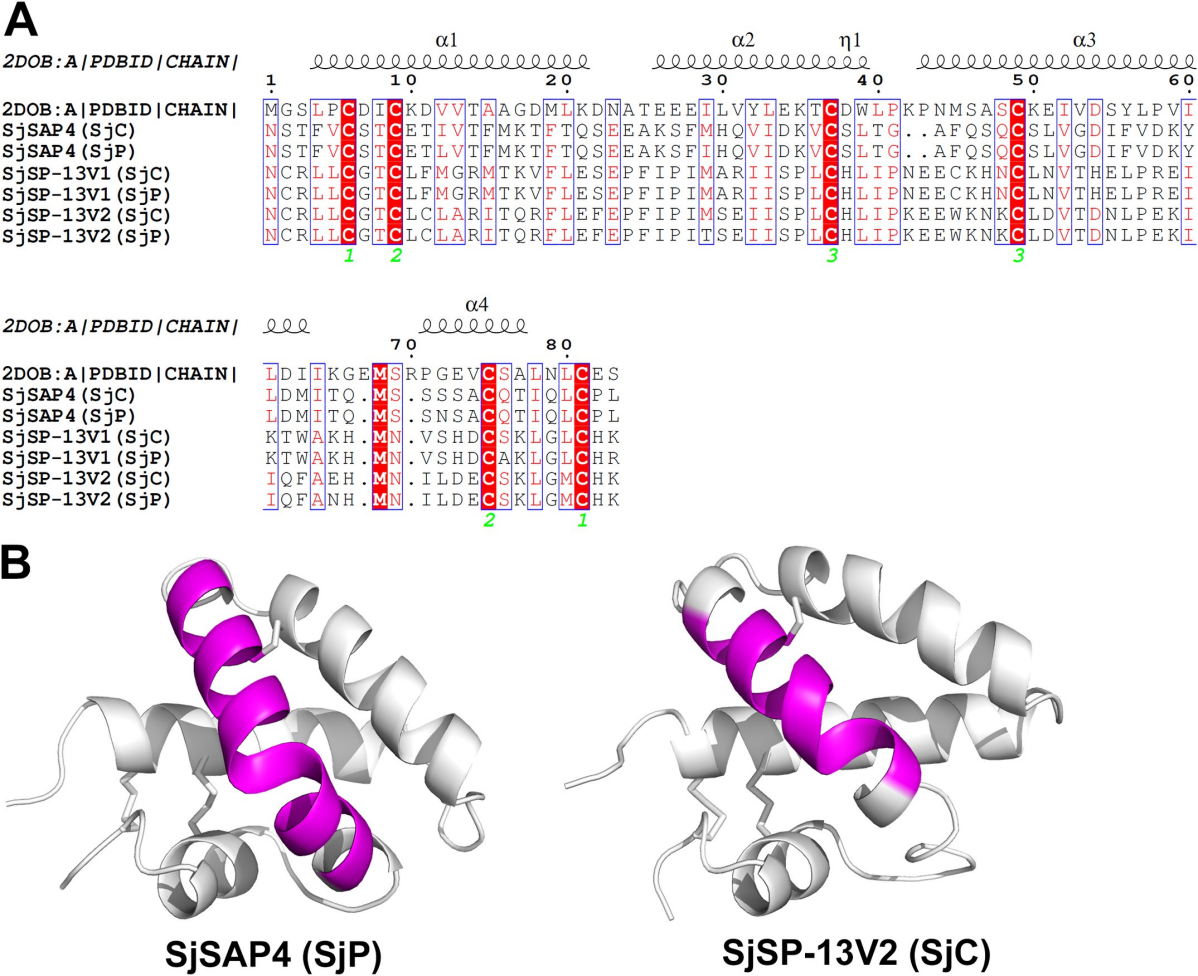
Multiple sequence alignment of the *Sj* SapB domains and epitope localization within the *Sj* SapB domains. **(A)** Structure-based sequence alignments of saposin domains of SjSAP4, SjSP-13 variant 1 (SjSP-13V1) and SjSP-13 variant 2 (SjSP-13V2). Secondary structure elements referring to the structure of human saposin A (PDB code 2DOB) are indicated on the top of the alignment. **(B)** Visualization of immunogenic epitopes in the 3D structure of the SapB domain of SjSAP4 (SjP) and SjSP-13V2 (SjC). Both the core B-cell epitopes (-S^163^QCSLVGDIFVDKYLD^178^- on SjSAP4 and -K^80^CLDVTDNLPE^90^- on SjSP-13V2) (indicated in magenta), are located in the third α-helix of the SapB domains.

The peptide sequences of the saposin domain of SjSAP4, SjSP-13 variant 1 (SjSP-13V1) and SjSP-13 variant 2 (SjSP-13V2) were aligned with homologous sequences of human saposin A (Fig 3A). The alignment showed that all domains are comprised of four α-helices with a typical characteristic that six conserved cysteine residues form three disulfide bonds. The three-dimensional structures of the SapB domain of SjSAP4 (SjP) and SjSP-13V2 were predicted by the Phyre 2.0 online severer. The core epitope (S^163^QCSLVGDIFVDKYLD^178^) is located in the third α-helix of the SapB domain of SjSAP4, similar to the known α-helix B-cell epitope (K^80^CLDVTDNLPE^90^) [30] within the SapB domain of SjSP-13V2 (Fig 3A and 3B).

### Structural analysis of SjSAP4 and electrostatic characteristics on the surface of the core epitope

**Fig 4:**
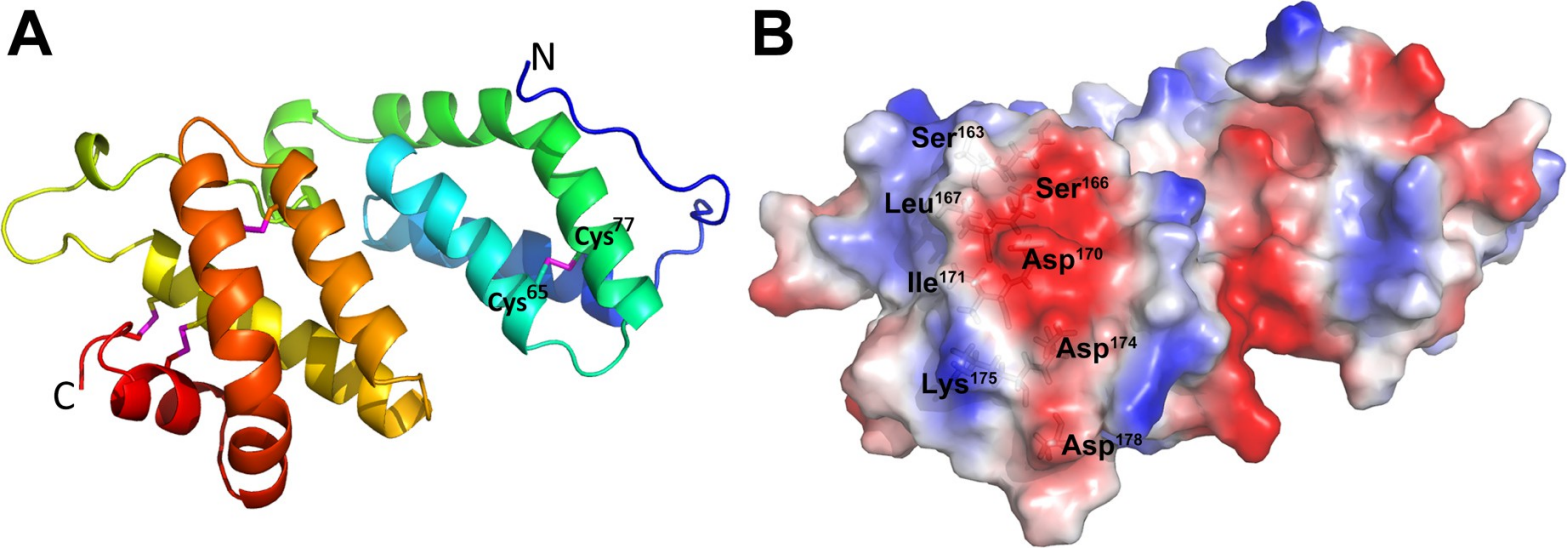
The three-dimensional structure of SjSAP4 and surface electrostatic contact potential analysis. **(A)** The predicted 3D structure of SjSAP4 (without signal peptide) colored in rainbow colors, shows an α-helix enriched region at the N-terminus, a disordered loop in the middle region, and a Sap-B domain at the C-terminus. The pink sticks indicate disulfide bonds formed between cysteine residues. **(B)** Electrostatic contact potential analysis showing the surface of the core epitope of SjSAP4 presents as a wavy plane with two hydrophobic AA in the center surrounded by polar and charged AA. Positively charged and negatively charged residues are colored in blue and red, respectively, whereas neutral residues are colored in white.

The structure of SjSAP4 (without signal peptide) was further predicted by the online algorithm Robetta. Structural analysis revealed that the protein has four α-helices within the SapB domain and non-saposin domain region, respectively, with a loop linking the two α-helix enriched regions (Fig 4A). In addition to the three disulfide bonds formed by the six conserved cysteines within the SjSAP4 SapB domain, another disulfide bond was formed between Cys^65^ and Cys^77^ at the N-terminal non-SapB domain region (Fig 4A). Electrostatic contact potential for the protein was generated by PyMOL using a charge-smoothed surface. This showed that the surface of the core epitope formed is a wavy plane for antibody docking, with two large neutral hydroponic residues (Leu^167^ and Ile^171^) in the center surrounded by polar AA (Ser^163^ and Ser^166^) and charged AA (Asp^170^, Asp^174^, Lys^175^ and Asp^178^) (Fig 4B).

### Diagnostic performance of the SjSAP4-, SjSAP4-Peptide-, SjSP-13V2-Peptide- and Sj23-Peptide-ELISAs

**Fig 5:**
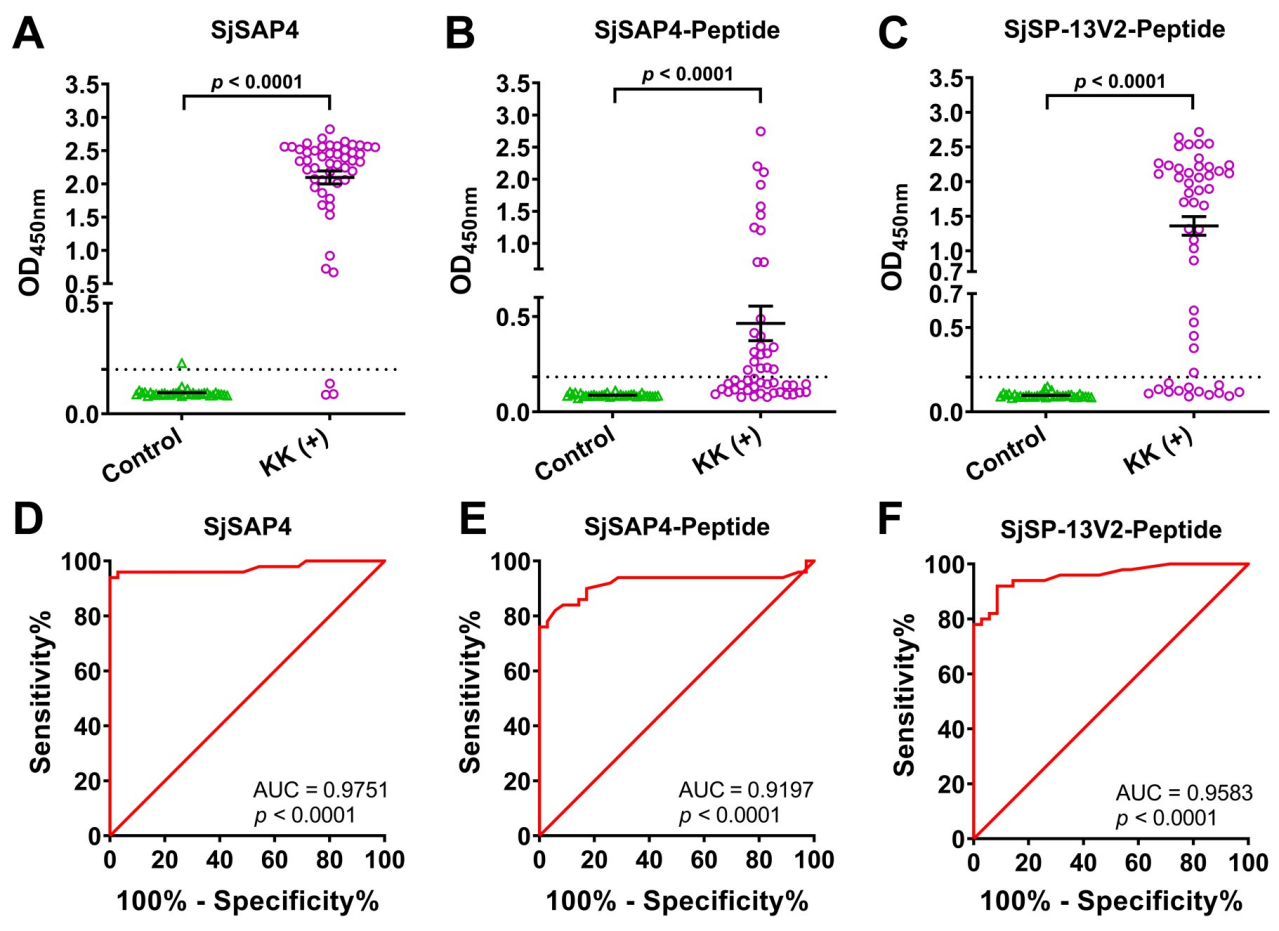
Diagnostic performance of the SjSAP4-, SjSAP4-Peptide- and SjSP-13V2-Peptide-ELISA. **(A-C)** Scatter plots showing the IgG responses to SjSAP4, SjSAP4-Peptide and SjSP-13V2-Peptide, respectively, in healthy controls (n = 35) and KK-positives (n = 50). Data were analyzed using a Mann Whitney *U*-test. **(D-F)** Receiver operating characteristic curve (ROC) analysis was performed for the SjSAP4-, SjSAP4-Peptide- and SjSP-13V2-Peptide-ELISA, respectively.

A 23-AA peptide (TSFHCCGVKGPDDYKGNVPASCK) from the large hydrophilic domain of Sj23 (Sj23-LHD), a 27-AA peptide (EWKNKCLDVTDNLPEKIIQFANHMNIL) from SjSP-13V2 and a 20-AA peptide (KQSQCSLVGDIFVDKYLDMK) from SjSAP4 were selected for diagnostic comparison. The IgG antibody levels against SjSAP4, the SjSAP4 peptide and SjPSP13-V2 peptide in KK-positives (n = 50) were significantly higher than uninfected healthy controls (n = 35) (*P* < 0.0001) (Fig 5A–5C); however, there was no difference in the IgG levels in the Sj23-Peptide-ELISA between the two groups (S2 Fig). The ROC curve analysis for discriminating the KK-positives from the controls yielded AUC values of 0.9751, 0.9197, and 0.9583 for the SjSAP4-, SjSAP4-Peptide- and SjSP-13V2-Peptide-ELISA, respectively (*P*< 0.0001 in all the assays) (Fig 5D–5F). When cut-off values were set, SjSAP4 showed a diagnostic sensitivity and specificity of 94.0% and 97.1%, respectively, and the SjSAP4-Peptide had a sensitivity of 46.0% and a specificity of 100%, while the SjSP-13V2-Peptide exhibited a sensitivity/specificity of 74.0%/100%.

### Diagnostic performance of the SjSAP4-Peptide + SjSP-13V2-Peptide-ELISA

**Fig 6:**
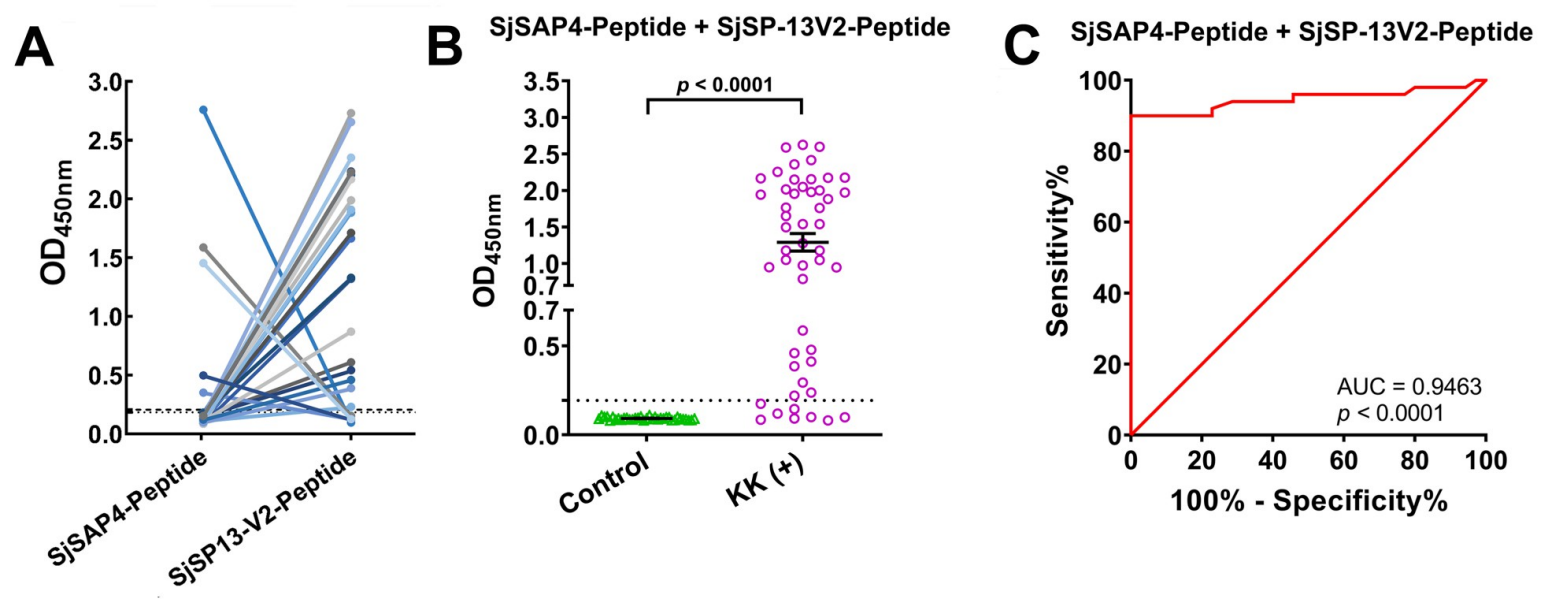
Complementary recognition of B-cell epitopes and diagnostic performance of the SjSAP4-Peptide + SjSP-13V2-Peptide-ELISA. **(A)** Complementary recognition of the SjSAP4-Peptide and SjSP-13V2-Peptide was observed in a subset of KK-positives (n = 25) by ELISA. **(B)** The specific IgG levels to the dual peptides (SjSAP4-Peptide + SjSP-13V2-Peptide) in healthy controls (n = 35) and KK-positives (n = 50) were evaluated by ELISA. Data were analyzed using a Mann Whiten *U*-test. (C) ROC analysis was performed for the SjSAP4-Peptide + SjSP-13V2-Peptide-ELISA.

Within the individual-peptide ELISA assays, we observed that of the 50 patients tested, twenty patients were SjSP-13V2-Peptide-ELISA positive but SjSAP4-Peptide-ELISA negative, while five of these were SjSAP4-Peptide-ELISA positive but SjSP-13V2-Peptide-ELISA negative (Fig 6A), indicating a differential recognition of B-cell epitopes among the schistosomiasis patients. We thus speculated whether the diagnostic performance could be improved if the two peptides were tested simultaneously. A dual-peptide ELISA (SjSAP4-Peptide + SjSP-13V2-Peptide-ELISA) was then developed and evaluated using sera from the same patient cohort. Compared to the healthy controls (n = 35), the KK-positives (n = 50) showed a significantly higher IgG levels against the dual peptides, SjSAP4-Peptide + SjSP-13V2-Peptide (*P* < 0.0001) (Fig 6B), with an AUC value of 0.9463 (*P* < 0.0001) (Fig 6C). With a cut-off OD value of 0.1936, the SjSAP4-Peptide + SjSP-13V2-Peptide-ELISA showed a diagnostic sensitivity/specificity of 84.0%/100%.

## Discussion

SjSAP4, a member of a multigene family of saposin-like proteins, has been demonstrated to be a highly immunogenic antigen along with other saposin proteins [23]. Previously, we developed an in-house ELISA based on the detection of specific SjSAP4 antibodies; it proved a cost-effective and an easy-to-use diagnostic tool with high sensitivity and specificity for large-scale cohort screening for individuals with schistosomiasis japonica in an endemic area of low-intensity in the Philippines [11, 26]. However, the recombinant SjSAP4 expressed in *E*. *coli* exhibits poor solubility in non-denatured solution, which may restrict or affect its broader application in other diagnostics formats, such as developing colloidal gold immunochromatography assay-based strips, which require a relatively higher concentration of soluble protein as target antigen. Although the solubility of recombinant SjSAP4 may be increased via other expression procedures such as eukaryotic cell-free or yeast expression systems, these protocols are usually cost-ineffective. In order to develop diagnostic antigens with more optimal physicochemical properties, such as size, solubility and heat-stability, one approach is to construct multi-epitope constructs based on B-cell epitopes identified from highly antigenic antigens. Subsequently, the resultant chimeric antigens can be evaluated for equivalent or even superior diagnostic performance compared to the original antigens, while at the same time retaining considerable solubility properties. In the current study, we identified a linear B-cell epitope on SjSAP4 and evaluated its potential as a component of a multi-epitope diagnostic antigen assay for the detection of schistosomiasis japonica.

Two linear B-cell epitopes within the SapB domain of another saposin protein, SjSP-13V2, were previously identified [30]. In the current study, by expressing 17 SjSAP4-derived peptides with 10-overlapped AA between any two adjacent peptides in the form of GST fusion proteins, only one peptide P15 was recognized in three out of five schistosomiasis-positive patient serum samples. Furthermore, the core epitope of P15 (S^163^QCSLVGDIFVDKYLD^178^), which is located in the third α-helix of the saposin-like domain, was determined by probing a panel of truncated peptides with human sera. The core epitope has four polar AA at the N-terminus and a preponderantly hydrophobic stretch (L^167^ –D^178^) at the C-terminus embedded with some charged AA. In addition, a B-cell epitope (K^80^CLDVTDNLPE^90^) is also located in the same region of the saposin domain of SjSP-13V2 (Fig 3). Notably, peptide P15, which forms an α-helix and is relative highly hydrophobic, was unfavorably predicted as a linear B-cell epitope by the prediction algorithms ABCPred [34] and BepiPred [35], which usually can predict continuous B-cell epitopes based on the physicochemical properties of a protein (e.g., hydrophilicity, solvent accessibility, flexibility, turns, polarity, antigenicity, and surface exposure) [39].

The performance of the SjSP-13V2-Peptide-, SjSAP4-Peptide- and SjSAP4-ELISAs in the diagnosis of schistosomiasis japonica was further evaluated. The SjSAP4-ELISA exhibited a sensitivity of 94.0% and a specificity of 97.1%, which was consistent with our previous observations [26]. When compared with the SjSAP4-ELISA, the sensitivity of the SjSAP4-Peptide-ELISA was reduced by half (from 94.0% to 46.0%), although it exhibited a specificity of 100%. In a previous study, a 15.5% reduction in sensitivity was observed when a SjSP-13-Peptide was compared with the full-length SjSP-13 by ELISA [30]. The considerable reduction in sensitivity of the SjSAP4-Peptide-ELISA can be explained by the following reasons: 1) The existence of discontinuous conformational B-cell epitopes on SjSAP4. The structural analysis we undertook (Fig 4A) indicated that the native SjSAP4 may form a solid conformation, which make it more prone to produce discontinuous epitopes. 2) The existence of weakly immunogenic B-cell epitopes on SjSAP4 (e.g., on the peptides P11 and PP1). 3) It has been suggested that the majority of B-cell epitopes range from 15 to 25 amino acids [39] or 15 ± 4 residues [40]; we thus cannot exclude the presence of additional linear B-cell epitopes that span over the 10-overlapped AA between the two adjacent peptides. 4) The nature of the synthesized peptide for testing. The initial synthesis of a 24-AA peptide -G^159^AFQSQCSLVGDIFVDKYLDMITQ^182^- and an 18-AA peptide -Q^162^SQCSLVGDIFVDKYLDM^179^- were unsuccessful due to the poor solubility of both peptides. Instead, a 20-AA peptide -KQSQCSLVGDIFVDKYLDMK-, in which an exogenous amino acid, lysine, was added to the ends of the 18-AA peptide Q^162^-M^179^ to increase solubility, was synthesized for testing in ELISA. Whether the two additional charged amino acids affected the peptide recognition by patient sera remains to be established.

In the current study, the SjSP-13V2-peptide (SjP) exhibited a sensitivity of 74.0%, which was consistent with its homologous peptide in SjC (76.7%) reported by Ma et al [30], and both showed a specificity of 100%. In addition, the sensitivity of the SjSP13-V2-peptide was higher than that of SjSAP4-peptide, which may be due to the peptide ‘-EWKNKCLDVTDNLPEKIIQFANHMNIL-’ being comprised of two B-cell epitopes, i.e., K^80^CLDVTDNLPE^90^ and E^90^KIIQFAN^97^ (E^90^KIIQFAE^97^ in SjC) [30]. Notably, the latter B-cell epitope, E^90^KIIQFAE^97^, which is a loop epitope, exhibited a stronger reactivity with patient sera than the former; i.e., of seven patient serum samples tested, the E^90^KIIQFAE^97^ epitope was recognized by six serum samples, while only two of the patient sera showed positive reactivity with K^80^CLDVTDNLPE^90^ [30].

The 23-AA peptide (-TSFHCCGVKGPDDYKGNVPASCK-) from Sj23-LHD, a homologous sequence to the B-cell epitope P14 of Sm23, which was originally identified using the murine schistosomiasis model [38], is not effectively recognised by the sera of schistosomiasis japonica patients. Indeed, immunogenicity elicited by Sj23-LHD during schistosome infection was shown to be higher in mice than in humans [26]. These observations suggest that humans may be unable to elicit antibodies against some specific epitopes that can be recognised by mice; in addition, schistosome infection intensity in humans is usually substantially lower than that in experimentally infected mice. Furthermore, the synthesized peptide may fail to mimic its native conformation, which is usually bent into a sub-loop structure due to the formation of disulfide bonds between the conserved cysteines, a typical characteristic of the large extracellular loop of tetraspanins [41].

Importantly, of the 50 KK-positive individuals tested in this study, twenty were SjSP-13V2-Peptide positive but SjSAP4-Peptide negative, while five were SjSAP4-Peptide positive but SjSP-13V2-Peptide negative. The individual differences in epitope recognition may be due to genetic polymorphisms in human HLA class II alleles. Notably, there is evidence that the HLA class II polymorphism influences alleles that play crucial roles in modulation of the hepatic immunopathology in hepatosplenic schistosomiasis [42–45]. Nevertheless, the complementary recognition of B-cell epitopes by the sera from different KK-positive individuals indicates that diagnostic sensitivity could be increased once multiple epitopes are formulated into chimeric antigens, thereby providing the basis for developing multi-epitope antigens for improved serological diagnosis of schistosomiasis. Indeed, the dual epitope-ELISA (SjSP-13V2-Peptide + SjSAP4-Peptide-ELISA) exhibited a higher sensitivity than the single epitope ELISAs (SjSP-13V2-Peptide-ELISA and SjSAP4-Peptide-ELISA).

There are a number of limitations to the current study. Firstly, the majority of linear B-cell epitopes range from 15 to 25 amino acids [39] or 15 ± 4 residues [40]. However, 10-AA overlapping peptides were used for the epitope mapping in the present study, which may miss linear B-cell epitopes that span over the 10-overlapped AA. A rational design of 20-mer peptides with an overlapping of 15-AA between any two adjacent peptides for mapping may help identify additional linear B-cell epitopes on the SjSAP4 protein. Secondly, bovines are the most important reservoirs driving the transmission of schistosomiasis japonica in endemic areas [46]. The current work did not evaluate the diagnostic performance of the SjSAP4-Peptide-ELISA and SjSP-13V2-Peptide + SjSAP4-Peptide-ELISA in *S*. *japonicum*-infected bovines. Although it does not affect the basis of this study, it is of importance to carry out such validation in the future. Thirdly, coating-efficiency of synthetic peptides is usually lower than that of full-length recombinant proteins and varies between peptides. Carbonate coating buffer is used for peptide coating in ELISA assays, but no coating-efficiency was assessed here for the three peptides tested. As the SjSAP4 peptide (20-mer) is shorter than the SjSP-13V2 peptide (27-mer), the low sensitivity of the SjSAP4-Peptide-ELISA may be partly caused by the relatively low binding efficiency of the peptide to the microplates used here.

## Conclusions

In the current study, a linear B-cell epitope (-S^163^QCSLVGDIFVDKYLD^178^-) was identified on a saposin protein, SjSAP4, an immunodominant antigen from *S*. *japonicum* through screening a series of GST-peptide fusion proteins. The diagnostic performance of the SjSAP4-Peptide was compared with an immunodominant peptide from SjSP-13V2 and the full-length SjSAP4 by testing human serum samples from infected individuals and healthy controls. Although the sensitivity of the SjSAP4-Peptide-ELISA was lower than that of the SjSAP4- and SjSP-13V2-Peptide-ELISAs, complementary recognition of B-cell epitopes was observed within a subset of the individual human sera tested. The dual-epitope ELISA (SjSP-13V2-Peptide + SjSAP4-Peptide-ELISA) showed a superior diagnostic performance than individual-epitope ELISAs. The study thus highlights the possibility that multi-epitope chimeric antigens with a diagnostic performance equivalent or superior to their parent antigens could be developed for the diagnosis of schistosomiasis through integrating immunogenic epitopes such as those from saposins (SjSP-13, SjSAP4 and SjSAP5) and non-saposin candidates [18].

## Supporting information

S1 Fig Prediction and validation of linear B-cell epitopes on SjSAP4. (**A**) PP1, PP2, and PP3 are potential B-cell epitope-containing peptides predicted by the online prediction methods BepiPred and ABCPred. PP4 is a combination of P10 and P11. **(B)** Western blot analysis showing that PP1 and PP4 were only weakly recognized by the serum sample from patient #2. M, Pre-stained protein ladder. Recombinant SjSAP4 protein was used as a positive control. All the serum samples from KK-positives were pre-incubated with purified GST protein.
(TIF)

S2 Fig No difference in the IgG response to SjS23-Peptide was observed between healthy controls (n = 35) and KK-positives (n = 50). Data were analyzed using a Mann Whitney *U*-test.
(TIF)

S1 Table Faecal egg burden of schistosomiasis japonica patients determined by the KK method. (XLSX)

S2 Table Primers used for molecular cloning in this study. (XLSX)
