# Supplementary figures and images for: Identification of a linear B-cell epitope on the Schistosoma japonicum saposin protein, SjSAP4: Potential as a component of a multi-epitope diagnostic assay
Source: PLoS Negl Trop Dis. 2022 Jul 11;16(7):e0010619. doi: 10.1371/journal.pntd.0010619 (PMC9302751; doi:10.1371/journal.pntd.0010619)

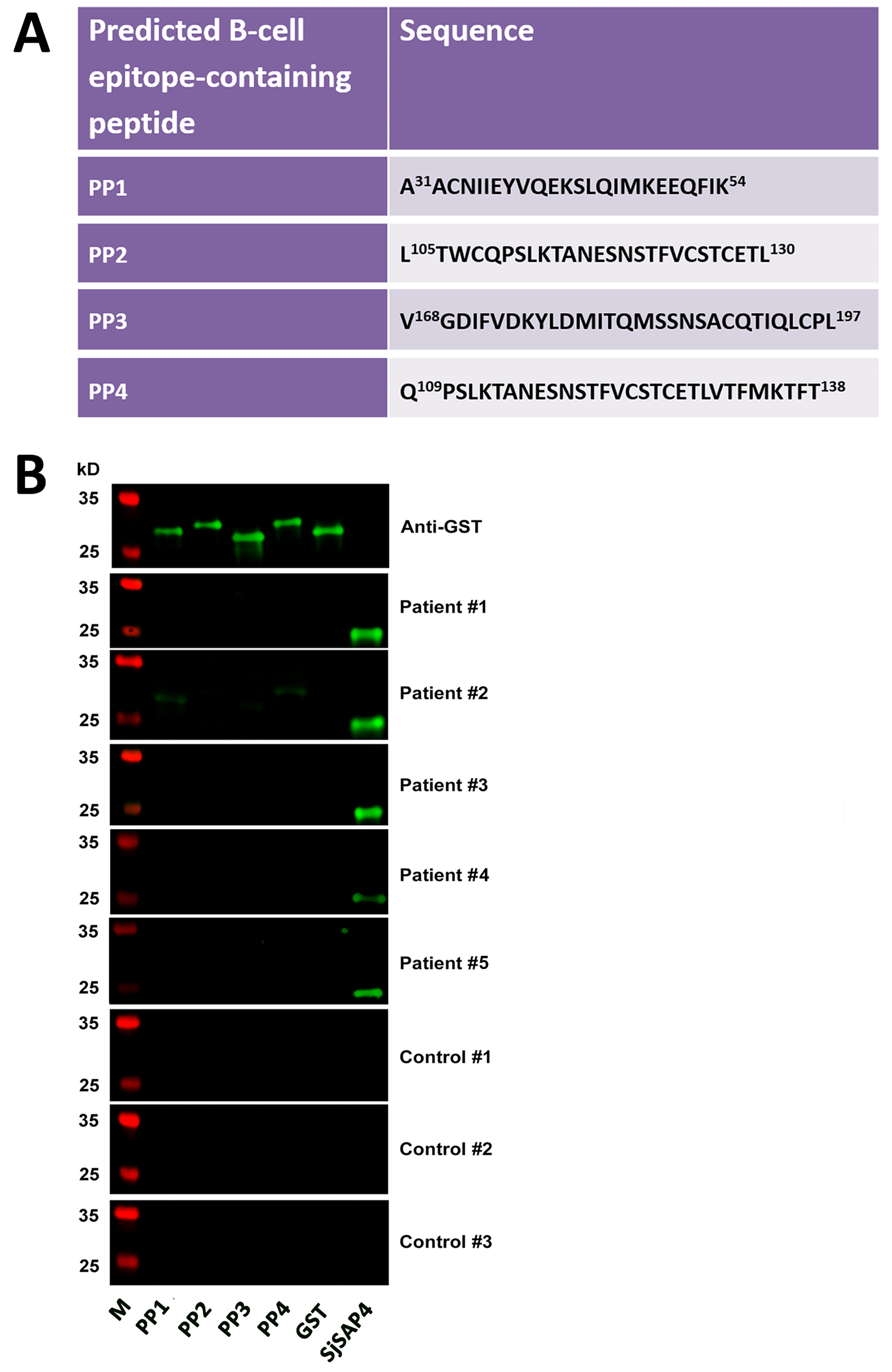

Supplement: S1 Fig — (A) PP1, PP2, and PP3 are potential B-cell epitope-containing peptides predicted by the online prediction methods BepiPred and ABCPred. PP4 is a combination of P10 and P11. (B) Western blot analysis showing that PP1 and PP4 were only weakly recognized by the serum sample from patient #2. M, Pre-stained protein ladder. Recombinant SjSAP4 protein was used as a positive control. All the serum samples from KK-positives were pre-incubated with purified GST protein. (TIF) [file pntd.0010619.s001.tif]

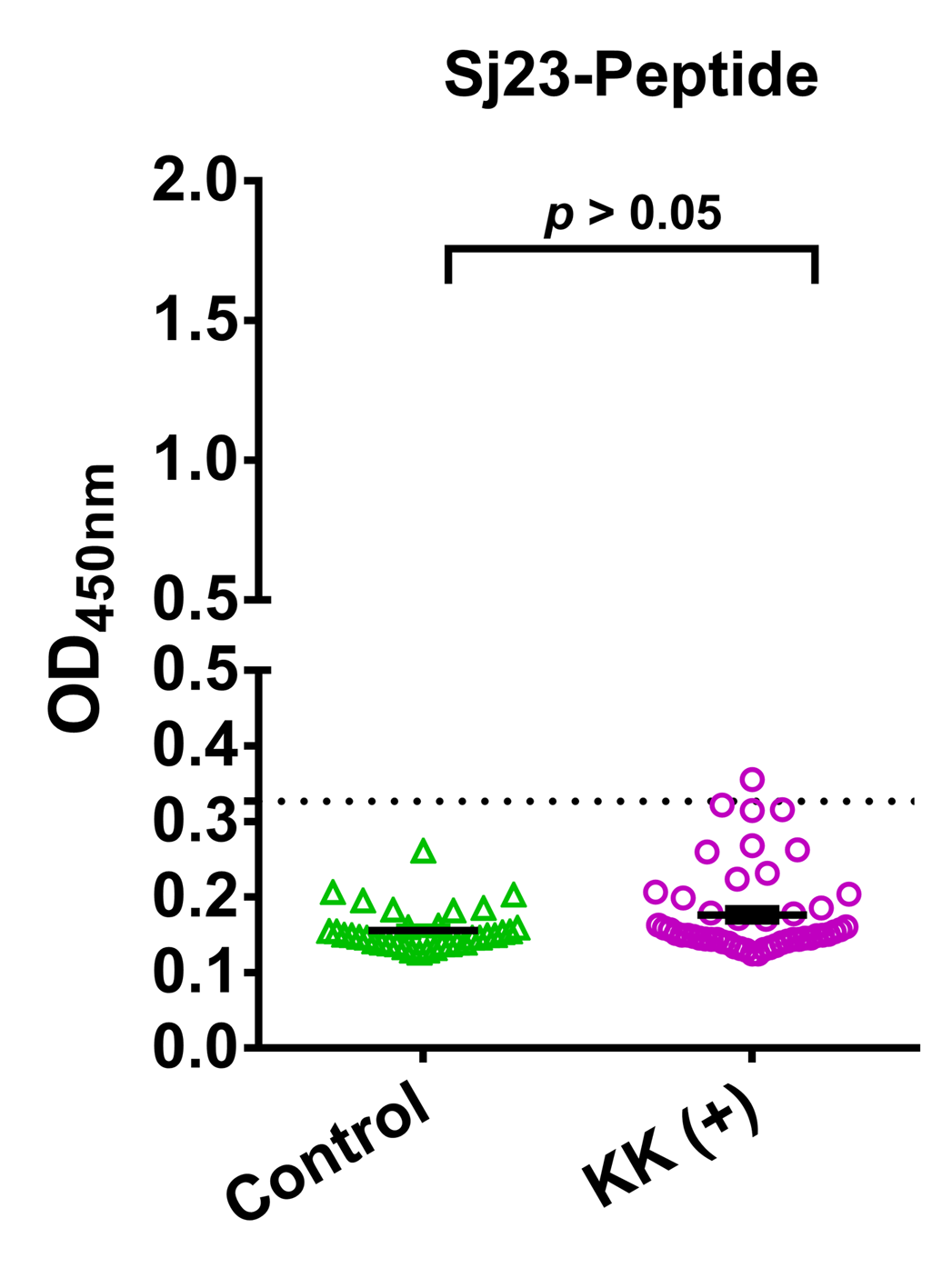

Supplement: S2 Fig — Data were analyzed using a Mann Whitney U-test. (TIF) [file pntd.0010619.s002.tif]
